# Impact of different mosquito collection methods on indicators of *Anopheles* malaria vectors in Uganda

**DOI:** 10.1186/s12936-022-04413-1

**Published:** 2022-12-19

**Authors:** Henry Ddumba Mawejje, Jackson R. Asiimwe, Patrick Kyagamba, Moses R. Kamya, Philip J. Rosenthal, Jo Lines, Grant Dorsey, Sarah G. Staedke

**Affiliations:** 1 Infectious Diseases Research Collaboration, Kampala, Uganda; 2 London School of Hygiene and Tropical Medicine, London, UK; 3 Department of Medicine, Makerere University College of Health Sciences, Kampala, Uganda; 4 Department of Medicine, University of California, San Francisco, USA

**Keywords:** *Anopheles*, Human landing catches, CDC light trap, Prokopack aspirators, Pit trap

## Abstract

**Background:**

Methods used to sample mosquitoes are important to consider when estimating entomologic metrics. Human landing catches (HLCs) are considered the gold standard for collecting malaria vectors. However, HLCs are labour intensive, can expose collectors to transmission risk, and are difficult to implement at scale. This study compared alternative methods to HLCs for collecting Anopheles mosquitoes in eastern Uganda.

**Methods:**

Between June and November 2021, mosquitoes were collected from randomly selected households in three parishes in Tororo and Busia districts. Mosquitoes were collected indoors and outdoors using HLCs in 16 households every 4 weeks. Additional collections were done indoors with prokopack aspirators, and outdoors with pit traps, in these 16 households every 2 weeks. CDC light trap collections were done indoors in 80 households every 4 weeks. Female *Anopheles* mosquitoes were identified morphologically and *Anopheles gambiae *sensu lato were speciated using PCR. *Plasmodium falciparum* sporozoite testing was done with ELISA.

**Results:**

Overall, 4,891 female *Anopheles* were collected, including 3,318 indoors and 1,573 outdoors. Compared to indoor HLCs, vector density (mosquitoes per unit collection) was lower using CDC light traps (4.24 vs 2.96, density ratio [DR] 0.70, 95% CIs 0.63–0.77, p < 0.001) and prokopacks (4.24 vs 1.82, DR 0.43, 95% CIs 0.37–0.49, p < 0.001). Sporozoite rates were similar between indoor methods, although precision was limited. Compared to outdoor HLCs, vector density was higher using pit trap collections (3.53 vs 6.43, DR 1.82, 95% CIs 1.61–2.05, p < 0.001), while the sporozoite rate was lower (0.018 vs 0.004, rate ratio [RR] 0.23, 95% CIs 0.07–0.75, p = 0.008). Prokopacks collected a higher proportion of *Anopheles funestus* (75.0%) than indoor HLCs (25.8%), while pit traps collected a higher proportion of *Anopheles arabiensis* (84.3%) than outdoor HLCs (36.9%).

**Conclusion:**

In this setting, the density and species of mosquitoes collected with alternative methods varied, reflecting the feeding and resting characteristics of the common vectors and the different collection approaches. These differences could impact on the accuracy of entomological indicators and estimates of malaria transmission, when using the alternative methods for sampling mosquitos, as compared to HLCs.

**Supplementary Information:**

The online version contains supplementary material available at 10.1186/s12936-022-04413-1.

## Background

Malaria remains a major public health concern globally, and particularly in sub-Saharan Africa, despite considerable effort to control it [1]. Uganda is typical of high burden countries in Africa and ranked third in number of malaria cases worldwide in 2021, contributing 5.4% of the global burden [1, 2]. *Plasmodium falciparum* accounts for 97% of malaria cases in Uganda [3, 4]. In Uganda and elsewhere in sub-Saharan Africa, the primary malaria vectors are *Anopheles gambiae *sensu stricto (*s.s*.), *Anopheles arabiensis* and *Anopheles funestus *sensu lato (*s.l*.) [1, 3, 5]. Deployment of vector control tools, including long-lasting insecticidal nets (LLINs) and indoor residual spraying (IRS) of insecticides, has been instrumental in reducing the burden of malaria, but the emergence and spread of insecticide resistance threatens the effectiveness of these measures [6]. Monitoring the impact of vector control tools through entomologic surveillance is essential to guide policy and programmes, but different sampling methods may influence entomologic outcome measures due to species-specific differences in the feeding and resting behaviours of *Anopheles* vectors. Moreover, the precision of the different collection methods varies, which may influence results [7–10].

Human landing catches (HLCs) are considered the ‘gold standard’ for monitoring human exposure to malaria mosquito vectors [11, 12]. HLCs involve overnight collection of mosquitoes from the exposed limbs of volunteers, using hand-held aspirators and torches; collections can be done both indoors and outdoors [12, 13]. HLCs provide a reliable estimate of key entomologic indicators including mosquito vector density, *Anopheles* species composition, sporozoite infection rate, and annual entomological inoculation rate (aEIR), defined as the number of infective bites per person per year [14, 15]. However, HLCs are expensive and labour intensive, and the positioning of collectors inside households overnight raises ethical issues, as does the intentional exposure of collectors to potentially infectious malaria vectors, even if prophylaxis is provided [7, 13]. These challenges have limited the widespread use of HLCs for entomological surveillance [16]. Alternative sampling methods include Centers for Disease Control (CDC) light traps and prokopack aspirators for indoor collections and pit traps for use outdoors [17–20] CDC light traps are attractive alternatives to HLC for indoor mosquito collection [10, 18, 21–23]. These traps use a light source to attract free-flying mosquitoes and a rotating fan to create suction pressure to trap mosquitoes in a collection cup [21]. Compared to HLCs, CDC light traps provided equivalent estimates for human biting rates [9, 23], *Anopheles* age structure [21], and sporozoite rates [23, 24], while the density of mosquitoes captured in CDC light traps was higher in some environments [24]. However, measurements using CDC light traps can vary with trap position and presence of human hosts in the house during collections, and may underestimate *Anopheles* species abundance [10, 25] or overestimate human biting rates and aEIR [7]. Moreover, CDC light traps have limited application outdoors [23], and may require two visits to households per collection [18, 23, 26].

Prokopack aspirators are another alternative to HLCs which target indoor resting adult mosquitoes. Prokopacks utilize a battery-powered lightweight motor unit connected to a mosquito collection cup, with an extendable arm to reach mosquitoes resting on ceilings. Mosquitoes are captured by the suction pressure created by an inbuilt fan [19, 27, 28]. Prokopack aspirators are relatively inexpensive and easy to use, and require only a single visit to the households per collection, which is attractive for large-scale vector surveillance [19]. However, in some settings, the density of vectors collected with prokopacks was lower than with indoor HLCs and CDC light traps, which is a potential disadvantage [28, 29].

Pit traps were developed in the 1940s and are the oldest method for collecting outdoor resting mosquitoes [20, 30, 31]. Pit traps involve digging artificial pit shelters approximately 5–6 ft deep under a shaded area, with cavities carved into the vertical sides of the pit to capture mosquito vectors resting outside human dwellings [20, 30]. Pit traps have been used to examine the impact of vector control interventions on vector density, species composition, human blood index and sporozoite infection rates [32]. Compared to HLCs, the density of mosquitoes captured in pit traps was higher [8, 33]. To further evaluate different mosquito collection methods both indoors and outdoors, this study compared four different methods to collect *Anopheles* vectors on key outcomes including vector density, species composition, sporozoite rate and aEIR. Indoors, prokopack aspirators and CDC light traps were compared to HLCs, and outdoors, pit traps were compared to HLCs.

## Methods

### Study sites

**Fig. 1:**
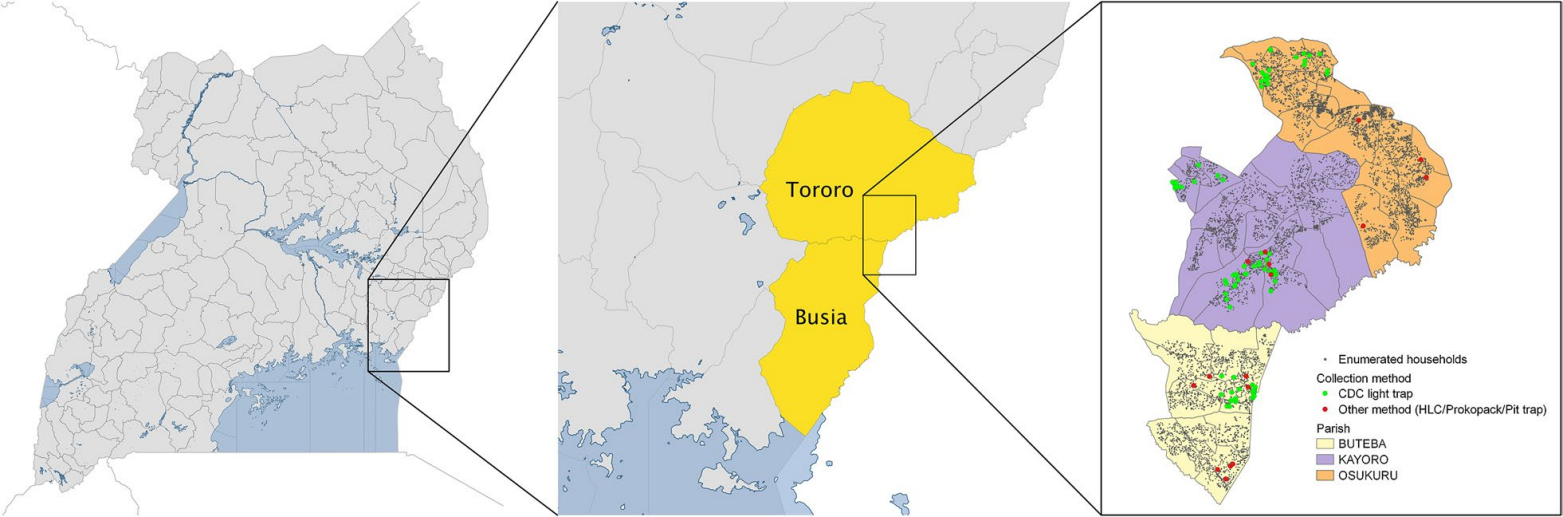
Map of study sites showing location of the 3 parishes including Buteba, Kayoro and Osukuru, in Busia and Tororo districts. The green dots highlight the positioning of the 80 border cohort households and the red dot show the position of the 16 households used for Human landing catches, prokopack and pit trap collections. Image modified from Nankabirwa et al. [39]

The study was conducted between June and November 2021 in Tororo and Busia districts. Both districts are in the Bukedi sub-region [4], in eastern Uganda bordering Kenya. The study area included Buteba parish in peri-urban Busia, and Kayoro and Osukuru parishes in rural Tororo (Fig. 1). These areas are characterized by low lying savannah plains, interspersed with bare rock and wetlands, and two annual rainfall peaks occurring between May–June and November–December [34]. Historically, Tororo district was a very high malaria transmission site with an aEIR measured at 562 infective bites per person per year in 2001 [35], and 125 in 2011–2012 [23]. Following implementation of regular rounds of IRS in 2014, combined with LLINs, which are delivered by the Ministry of Health every 3–4 years, malaria burden in Tororo reduced dramatically [36]. By 2019, the measured aEIR had dropped to 0.43 infective bites per person per year [36]. However, after five years of intensive vector control and sustained low-level transmission [37], a resurgence of malaria exceeding pre-IRS levels was documented in Tororo and other areas receiving IRS in 2020–2021 [38]. The etiology of the resurgence has not yet been established, but recent changes in the insecticide delivered by IRS is suspected [38]. In 2020–2021, coinciding with the mosquito sampling for this study, parasite prevalence in the study area was 19.5% by microscopy and 50.7% by qPCR, with no significant differences between Tororo and Busia [39]. In Tororo, the primary malaria vector species include *An. gambiae s.s*., *An. arabiensis* and *An. funestus s.l.* [23]. Following introduction of IRS, *An. arabiensis* became the predominant species [40]. More recently, coincident with the change in IRS insecticide, increases in both *An. gambiae s.s*. and *An. funestus* mosquito density have been observed in Tororo district (unpublished data). Busia is also a site of very high malaria transmission [41, 42], but unlike Tororo, Busia has received LLINs only (without IRS) for vector control. Malaria transmission patterns in Busia are stable and characteristic of a high transmission area [37, 41]. The dominant malaria vectors in Busia are *An. gambiae s.s.* and *An. funestus,* and to a lesser extent *An. arabiensis* [43]. In 2020–2021, the annual EIR was higher in Busia (108.2 infective bites/person/year) than in Tororo (59.0 in Osukuru parish vs 27.4 in Kayoro parish) [39].

### Households selected for entomological surveillance

Mosquito samples were collected under the PRISM (Program for Resistance, Immunology, Surveillance and Modeling of Malaria) Border Cohort study [39], initiated in August 2020 in three adjacent parishes (Fig. 1), including two parishes in Tororo district and one parish in Busia district. Prior to the study, all households in the study area were enumerated and mapped (n = 10,474), to generate a sampling frame for the study. The study area was stratified into three transmission areas based on parasite prevalence data. In August 2020, randomly selected households from the three transmission areas were approached and screened for eligibility. Households were enrolled into the cohort study if they met the following selection criteria: (1) at least two members less than 5 years of age, (2) no more than 7 permanent members currently residing, (3) no plans to move from the study area in the next 2 years, and (4) willingness to take part in entomological surveillance activities [39]. A total of 80 randomly selected households were enrolled, including 20 households in Busia, 30 houses from Kayoro, Tororo near the Busia border, and 30 houses from Kayoro and Osukuru, Tororo away from the Busia border. In all 80 households participating in the cohort study, mosquitoes were collected using CDC light traps every 14 days. An additional 16 households (8 from Busia and 8 from Tororo) not taking part in the cohort study were randomly selected from the enumeration database to participate in indoor and outdoor HLCs, which were conducted every 4 weeks [39]. For the purposes of this study, prokopack aspirator collections and pit trap collections were also done in the same 16 non-cohort households 1 week prior and one week after the HLCs. Data collected between June and October 2021 were included in this analysis, covering 6 rounds of HLCs (every 4 weeks), 12 rounds of prokopack aspirator and pit trap collections (every 2 weeks, 1 week before and after HLCs), and 6 rounds of CDC light trap collections (every 4 weeks, closest date to when HLCs were done). All participating households provided written informed consent before study activities were conducted.

### Mosquito collection methods

This study, aimed to evaluate different mosquito collection methods as compared to HLCs as the gold standard for both indoor and outdoor collections. Both HLCs and CDC light traps have been evaluated previously in this area [23], and prokopack aspirators were used in a large-scale trial conducted in 48 districts in Uganda [44]. Pit traps have not been evaluated in Uganda but provide an additional method for sampling outdoor resting mosquitoes [30].

#### Human-landing catches

HLC households were located  > 300 m from each other. To ensure comparability of results, four households were sampled per night for 4 consecutive nights in order to have the 16 households sampled within the same week for each 4 week interval. For the HLCs, four adult collectors were stationed at each house, with two indoors and two outdoors at a distance of at least 10 m. Indoor and outdoor collections were conducted from 18:00 h at dusk to 08:00 h in the morning, with hourly recordings of mosquitoes caught. A 10 min break was given for each hour of collection. Mosquito collectors used hand-held aspirators and torches to capture mosquitoes that landed on their exposed limbs. Collectors were rotated between sites and collection times to limit field collector bias. All mosquitoes collected were transferred to paper cups and transported for further processing.

#### CDC light trap collections

CDC light trap collections were conducted in all 80 households participating in the PRISM cohort study. Miniature CDC light traps (Model 512; John W. Hock Company, Gainesville, Florida, USA) were positioned 1 m above the floor at the foot end of a human occupied bed covered by a standard pyrethroid-only LLIN. CDC light traps were set in all rooms where household members sleep. Traps were set at 19:00 h and collected at 07:00 h the following morning. All mosquitoes collected in the light traps were stored individually for further processing.

#### Prokopack aspirators

Prokopack collections were conducted using a battery powered mosquito aspirator (InsectaZooka) [27] with a lightweight motor and suction cups for mosquito collection. The prokopack was connected to a 12 V battery, which was carried by the operator in a backpack to ease mobility. Prokopack collections were conducted a week before and the week following HLC sampling, ‘sandwiching’ HLCs to improve spatial comparison of mosquito density estimates. Prokopack collections were conducted on a single morning per household and scheduled not interfere with HLC collections. Resting mosquitoes were collected in the early morning hours (between 06.00 h and 08.00 h) while the temperature was cooler, to standardize collections and maximize yields. Two field workers spent at least 30 min inside each house, which was previously shown to be adequate in Uganda [44], and collected all mosquitoes resting on walls, on the ceiling, under tables and beds. Four houses were sampled each day, to ensure sampling of the 16 houses was done within the same week. All mosquitoes collected were transferred to paper cups and transported for further processing. Mosquitoes were transported using cool boxes to the study insectary, sorted and stored dry on desiccant (silica gel) for molecular analysis [23].

#### Pit trap collections

Mosquito pit traps were set up within 10–20 m of each of the 16 households where HLCs and prokopack collections were done. Pit trap collections were conducted every two weeks with the same schedule as prokopack collections, ‘sandwiching’ HLCs, between 06:00 h and 08:00 h. Four pit shelters were assessed at a time, so that 16 pit shelters were covered within the same week, matching the prokopack collections. Artificial pit shelters were dug 5–6 ft deep, under natural shade so that their openings (4 to 5 × 3 to 4ft) were shaded from above [20]. A suitable cover using locally sourced timber and thatch was placed partially over the pit trap entrance for shielding. About 2ft from the bottom of the pit trap, small un-baited cavities, about 30 cm deep were dug horizontally from each of the four sides of the pit. Mosquitoes were collected from these small cavities and from the wall of the pit itself. The pit traps were encircled with a thorn fence enclosure to prevent animals or children from falling into them or using them as toilets, as recommended by Muirhead-Thomson [20].

### Species identification and *Plasmodium falciparum* sporozoite ELISA

All female *Anopheles* mosquitoes collected were identified morphologically using previously described keys [45] and stored dry, individually in 1.5 ml tubes for further molecular analysis. Morphologically identified species included 3 groups: *An. funestus*, *An. gambiae s.l.*, and other *Anopheles*, which were primarily *Anopheles chrysti* considered to be non-malaria vectors [46]. All female *An. gambiae s.l.* collected by HLC (both indoor and outdoor), prokopack and pit traps were differentiated as *An. gambiae s.s.* and *An. arabiensis* using PCR [47]. For CDC light trap collections, a random sample of 60 *An. gambiae s.l.* per month was speciated due to resource limitations. *Plasmodium falciparum* sporozoite ELISA was conducted on all female *An. gambiae s.l.* and *An. funestus s.l.* collected by HLC, prokopack, CDC light traps, and pit traps, using the protocol developed by Wirtz et al. [48], which has previously been used in Uganda [23, 34, 39]. *Anopheles funestus s.l.* were only identified morphologically due to resource limitations.

### Statistical analysis

Vector density was defined as the total number of female *Anopheles* mosquitoes collected divided by the total number of collections done per method and expressed as the average number of mosquitoes per day for each method. The sporozoite rate was defined as the number of female *Anopheles* mosquitoes testing positive using ELISA divided by the total number tested. The aEIR was expressed as a product of daily vector density and the sporozoite rate multiplied by 365 days per year [23, 49]. Analyses were done using Stata (version 14.2, Stata Corp, College Station, TX, USA). For all analyses, data were collapsed for each collection method across the entire collection period. For all measures of association, data were stratified by whether collections methods were indoor or outdoor, and HLCs were considered the reference group. Associations between collection methods and vector density or aEIR were made using a negative binomial regression model with the number of collections included as an offset and associations expressed as the density ratio (DR) or incident rate ratio (IRR), respectively. Associations between collection methods and sporozoite rates were made using the Chi-squared or Fisher’s exact test. A two-sided p < 0.05 was considered statistically significant.

### Ethical approval

For all methods, a written informed consent was obtained from household heads or their designate before mosquito collection could commence. HLCs included additional consenting of the mosquito collectors. Ethical approval was obtained from Makerere University School of Medicine Research and Ethics Committee (SOMREC), the Uganda National Council of Science and Technology (UNCST), the London School of Hygiene and Tropical Medicine Research and Ethics Committee and the University of California, San Francisco Committee on Human Research.

## Results

### Mosquito collection

A total of 4,891 female *Anopheles* were collected, including 3,318 indoors and 1,573 outdoors (Table 1). For indoor collections, most mosquitoes were collected using CDC light traps (2,562), while outdoors, the majority were collected using pit traps (1,234). Of the 3,313 mosquitoes captured indoors that were tested for sporozoites, 43 were positive, including 6 of 407 collected using HLCs (4 *An. gambiae s.s.*, 1 *An. arabiensis* and 1 *An. funestus*) and 6 of 349 collected with prokopack aspirators (1 *An. arabiensis* and 5 *An. funestus*). Of the 2,557 mosquitoes collected using CDC light traps that were tested for sporozoites, 31 were positive, however, due to the way these data were collected it was not possible to assign sporozoite positivity to the species level. Of the 1,573 mosquitoes captured outdoors, 11 were positive for sporozoites, including 6 of 339 collected using HLCs (1 *An. arabiensis* and 5 *An. funestus*) and 5 of 1,234 collected using pit traps (1 *An. gambiae s.s.*, 4 *An. arabiensis*).

**Table 1:** Female *Anopheles* mosquito collections (*An. gambiae* s.l. and *An. funestus* s.l.) by different methods

Collection method	Sampled HHs	Total collections	Total *Anopheles* collected	Total number of *Anopheles* tested for sporozoites (number sporozoite positive)				
				All *Anopheles*	*An. gambiae* s.s	*An. arabiensis*	*An. funestus*	Other *Anopheles*
Indoor								
HLC	16	96	407	407 (6)	74 (4)	203 (1)	105 (1)	25 (0)
CDC LT	80	867	2562	2557 (31)	798 (N/A)	813 (N/A)	891 (N/A)	60 (N/A)
Prokopack	16	192	349	349 (6)	59 (0)	26 (1)	262 (5)	2 (0)
Outdoor								
HLC	16	96	339	339 (6)	62 (0)	125 (1)	117 (5)	35 (0)
Pit trap	16	192	1234	1234 (5)	49 (1)	1040 (4)	123 (0)	22 (0)

### Species composition

**Fig. 2:**
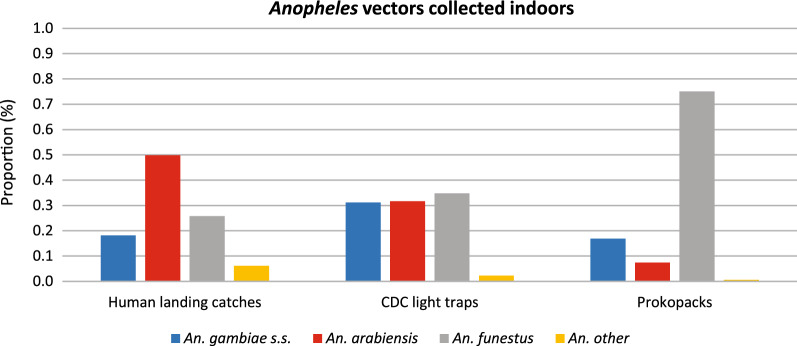
*Anopheles* vectors collected indoors using human landing catches (HLC), prokopack aspirators and CDC Light traps. The bars depict *Anopheles* mosquito species including *An. gambiae s.s.* (blue bar), *An. arabiensis* (red bar), *An. funestus* (grey bar) and other *Anopheles* (orange bar)

**Fig. 3:**
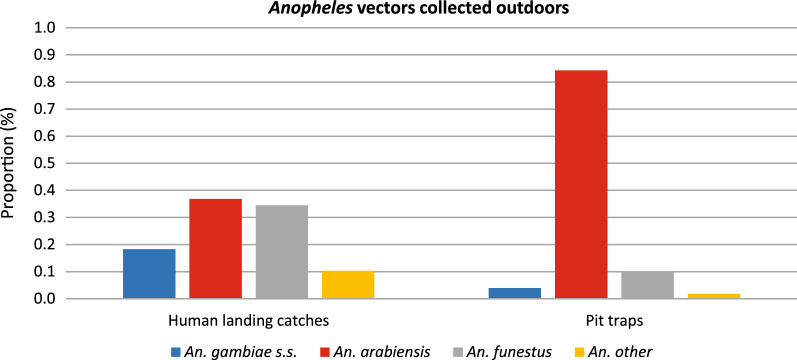
*Anopheles* vectors collected outdoors using human landing catches (HLC), and Pit traps. The bars depict *Anopheles* mosquito species including *An. gambiae s.s.* (blue bar), *An. arabiensis* (red bar), *An. funestus* (grey bar) and other *Anopheles* (orange bar)

The dominant species of *Anopheles* captured varied by whether collections were done indoors or outdoors and the method of collection used. All three main vectors were collected using indoor HLCs (Fig. 2), with *An. arabiensis* dominating (49.9%). Using CDC light traps, all three main vectors were collected in fairly similar proportions (ranges 31.1% to 34.8%). In contrast, using prokopack aspirators, a higher proportion of An. *funestus* were collected (75.1%). Outdoors, HLCs captured all three main vectors (Fig. 3), with *An. arabiensis* (36.9%) and *An. funestus* (34.5%) dominating. However, pit traps captured a higher proportion of *An. arabiensis* (84.3%).

### Measures of association between method of collection and key entomologic indicators

Compared to indoor HLCs, the density of mosquito vectors collected was lower using both CDC light traps (4.24 vs 2.96, DR 0.70, 95% CIs 0.63–0.77, p < 0.001) and prokopack aspirators (4.24 vs 1.82, density ratio [DR] 0.43, 95% CIs 0.37–0.49, p < 0.001). *Plasmodium falciparum* sporozoite rates were similar between the three indoor collection methods, although precision was limited due to the low numbers of sporozoites that were detected, especially using prokopack and HLC. Overall, the aEIR using CDC light traps or prokopack aspirators was approximately half what was estimated using indoor HLCs, however these differences did not achieve statistical significance (Table 2). In contrast, compared to outdoor HLCs, vector density was higher using pit trap collections (3.53 vs 6.43, DR 1.82, 95% CIs 1.61–2.05, p < 0.001), while the sporozoite rate was lower (0.018 vs 0.004, DR 0.23, 95% CIs 0.07–0.75, p = 0.008). Overall, the aEIR using pit traps was less than half what was estimated using outdoor HLCs (22.81 vs. 9.51, IRR = 0.42, 95% 0.13–1.37), although this difference did not reach statistical significance (Table 2).

**Table 2:** Measures of association between method of collection and vector density, sporozoite rate and aEIR in female *Anopheles* mosquitoes

Collection method	Vector density	DR (95% CI)	P value	Sporozoite rate	RR (95% CI)	P value	aEIR	IRR (95% CI)	P value
Indoor									
HLC	4.24	Reference	–	0.015	Reference	–	22.81	Reference	–
CDC LT	2.96	0.70 (0.63–0.77)	< 0.001	0.012	0.82 (0.34–1.96)	0.66	13.08	0.57 (0.24–1.37)	0.21
Prokopack	1.82	0.43 (0.37–0.49)	< 0.001	0.017	1.17 (0.38–3.58)	0.79	11.41	0.50 (0.16–1.55)	0.23
Outdoor									
HLC	3.53	Reference	–	0.018	Reference	–	22.81	Reference	–
Pit trap	6.43	1.82 (1.61–2.05)	< 0.001	0.004	0.23 (0.07–0.75)	0.008	9.51	0.42 (0.13–1.37)	0.15

### Species-specific vector density and sporozoite rates, by method of collection

Compared to indoor HLCs, the density of *An. arabiensis* was significantly lower using CDC light traps (0.94 vs 2.11, DR 0.44, 95% CIs 0.38–0.52, p < 0.001); but no significant differences in vector density of *An. gambiae s.s.* or *An. funestus* were observed when CDC light traps and indoor HLCs were compared (Table 3). The density of *An. gambiae s.s.* and *An. arabiensis* collected using prokopack aspirators were significantly lower than with indoor HLCs (0.31 vs 0.77, DR 0.40, 95% CIs 0.28–0.56, p < 0.001; 0.14 vs 2.11, DR 0.06, 95% CIs 0.04–0.10, p < 0.001); for *An. funestus*, vector density was higher using prokopack aspirators than HLCs, but this difference was not statistically significant (Table 3). No differences in sporozoite rates were observed for *An. gambiae s.s.*, *An. arabiensis* or *An. funestus* when mosquitoes collected indoors using prokopack aspirators were compared to indoor HLCs (Additional file 1: Table S1).

**Table 3:** Measures of association between method of collection and vector density, stratified by species

	*Anopheles gambiae s.s*			*Anopheles arabiensis*			*Anopheles funestus*		
	Vector density	DR (95% CI)	P value	Vector density	DR (95% CI)	P value	Vector density	DR (95% CI)	P value
Indoor									
HLC	0.77	Reference	–	2.11	Reference	–	1.09	Reference	–
CDC LT	0.92	1.19 (0.94–1.52)	0.14	0.94	0.44 (0.38–0.52)	< 0.001	1.03	0.94 (0.77–1.15)	0.55
Prokopack	0.31	0.40 (0.28–0.56)	< 0.001	0.14	0.06 (0.04–0.10)	< 0.001	1.36	1.25 (0.99–1.56)	0.06
Outdoor									
HLC	0.65	Reference	–	1.30	Reference	–	1.22	Reference	–
Pit trap	0.26	0.40 (0.27–0.57)	< 0.001	5.42	4.16 (3.46–5.01)	< 0.001	0.64	0.53 (0.41–0.68)	< 0.001

The densities of *An. gambiae s.s.* and *An. funestus* collected using pit traps were lower than with outdoor HLCs (0.26 vs 0.65, DR 0.40, 95% CIs 0.27–0.57, p < 0.001; 0.64 vs 1.22, DR 0.53, 95% CIs 0.41–0.68, p < 0.001); for *An. funestus*, vector density was significantly higher using pit traps than HLCs (5.42 vs 1.30, DR 4.16, 95% CIs 3.46–5.01, p < 0.001; Table 3). No differences in sporozoite rates were observed for *An. gambiae s.s.* or *An. arabiensis* when mosquitoes collected outdoors using pit traps were compared to outdoor HLCs; however, for *An. funestus* the sporozoite rate in mosquitoes collected using pit traps was significantly lower than in those collected by outdoor HLCs (sporozoite rate 0.000 vs 0.043; 95% CIs 0.043 (0.0158–0.1018), fisher exact p = 0.03) (Additional file 1: Table S1).

## Discussion

Human landing catches, considered the gold standard for collecting host-seeking *Anopheles* indoors and outdoors are challenging to use on a large scale [7, 9, 13]. In this study, CDC light traps and prokopack aspirators were compared to HLCs for indoor mosquito collection, and pit traps were compared to outdoor HLCs. The density of *Anopheles* vectors collected indoors was 30% lower with CDC light traps and 57% lower with prokopacks as compared to HLCs. Sporozoite rates and aEIRs were not significantly different between the 3 indoor collection methods but the precision of these comparisons was limited by the low sporozoite rate. The relative species composition was similar between indoor HLCs and CDC light traps, but prokopacks, which only collected mosquitoes resting in the morning indoors, captured a higher proportion of *An. funestus* compared to indoor HLCs. Given these findings, CDC light traps provided a reasonable alternative to indoor HLCs, but prokopacks may not provide an accurate sampling of mosquitoes responsible for malaria transmission. Outdoors, the density of *Anopheles* vectors collected via pit traps was significantly higher than HLCs, however, sporozoite rates were significantly lower and a higher proportion of *An. arabiensis* were collected. Pit traps could be a useful alternative to HLCs for simply sampling outdoor resting mosquitoes, but provided less accurate estimates of measures of transmission intensity [8, 33]. In this setting, the density and species of mosquitoes collected with alternative methods varied, reflecting the feeding and resting characteristics of the common vectors and the different collection approaches, which impacted on the entomological indicators and estimates of malaria transmission.

CDC light traps are the most common alternative to HLCs for collection of indoor resting *Anopheles* [10, 21, 23]. Overall, CDC light traps are mechanical, less intrusive, non-exposure and efficient tools that are relatively simple to use in field settings, permitting overnight collection of mosquitoes [23]. In this study, CDC light traps collected modestly fewer *An. arabiensis* compared to HLCs indoors, however there was no significant difference in vector density for both *An. gambiae s.s.* and *An. funestus* when compared to HLCs. Similar observations were reported by Briet et al., [10]; where the relative sampling efficiency of CDC light traps for *Anopheles* vectors was comparable to HLCs indoors. Notably, Briet et al., also observed that the relative sampling efficiency for CDC light traps was greater for *An. funestus s.l.* compared to *An. gambiae s.l.* [10]. In several observations from sub-Saharan Africa, CDC light traps collected equivalent or higher numbers of *Anopheles* compared to HLCs [10, 21, 23] and were used as reliable alternatives for estimating sporozoite infection rates and EIR [23]. However, early findings from Kenya by Mbogo et al., showed that CDC light traps underestimated the abundance of *An. gambiae s.l.* [25]. In examining mosquito sampling techniques and their reliability, including HLCs, CDC light traps and odour-baited traps, Mboera et al., reported an overestimation of EIR in CDC light traps arising from very high vector densities [7]. CDC light traps may not have universal appeal, as observed in Bioko Island, where this method did not reliably estimate mosquito biting rates [26]. Differences in vector density, species composition and sporozoite infection rates have been observed with CDC light traps in different settings, showing distinct geographical patterns but largely with a positive correlation in *Anopheles* vector density to indoor HLCs [10, 18, 23]. Differences in *Anopheles* vector density, species composition and sporozoite infection rates were observed in response to changes in CDC light source, trap position, collection time and presence or absence of a human bait [18, 25, 50, 51]. Limitations notwithstanding, CDC light traps collected similar vector densities to indoor HLCs for highly anthropophilic vectors; *An. gambiae s.s.* and *An. funestus.* In addition, CDC light traps have been shown to provide reliable estimates for mosquito vector density in comparison to HLCs with increase in number of collection nights, making this tool suitable for longitudinal entomological surveillance [10, 23]. The recent deployment of solar-recharged CDC light traps in estimating *Anopheles* vector density, makes this tool an even more attractive alternative to HLCs in resource limited settings [52].

Prokopack aspirators are a relatively new tool for indoor mosquito collection [29]. Prokopacks are battery powered, light-weight motor units that collect indoor resting and free-flying mosquitoes using suction pressure [27]. Prokopack aspirators in this study collected significantly fewer mosquitoes indoors compared to HLCs, with significantly lower vector density for both *An. gambiae s.s.* and *An. arabiensis*. Comparison of prokopack aspirators with HLCs in coastal Kenya showed that prokopacks collected more *Culex quinquefasciatus* and other culicines than *Anopheles* vectors [29]. This finding, however, may have been influenced by the low density of *Anopheles* mosquitoes in the population sampled. Studies in Tanzania and Eritrea demonstrated the utility of prokopacks in estimating *Anopheles* vector density indoors, pre and post vector control interventions [28, 53, 54]. Prokopack aspirator collections provide an efficient mosquito collection technique operated by a single individual, requiring only 15–30 min in the household during a single visit, making prokopacks an attractive alternative to HLCs and a scalable tool for sampling indoor resisting mosquitoes [44]. In this study relatively more *An. funestus* were collected with prokopack aspirators compared to indoor HLCs. In contrast, prokopack collections across 48 districts in Uganda by Lynd et al., yielded significantly more *An. gambiae s.s.* than *An. funestus* [44]. Prokopack aspirators have been shown to be very effective in cross-sectional studies that require a snapshot assessment of *Anopheles* species composition, sporozoite infection rates and insecticide resistance variants [44].

Pit traps have been used for outdoor mosquito collections for over half a century [31]. Pit traps involve utilization of artificial pit shelters dug in the ground for collection of outdoor resting mosquitoes [20]. Comparison of pit traps with outdoor HLCs, showed significantly higher *Anopheles* vector density, albeit with significantly lower sporozoite infection rates. In addition, significantly more *An. arabiensis* were collected with pit traps outdoors compared to HLCs. However, significantly fewer *An. gambiae s.s.* and *An. funestus* were collected in the pit traps compared to outdoor HLCs. Pit traps have been used for assessment of outdoor resting mosquitoes, estimates of mosquito gonotrophic cycles, sporozoite infection and EIR [30]. In this study, pit traps mainly caught *An. arabiensis* similar to observations made in Moshi, Tanzania [33] and Konso, southern Ethiopia [8]. Pit traps provide a stationary outdoor mosquito trap that can be used for prolonged periods with limited maintenance [20]. However, the stationary nature of pit traps is also a major limitation to the scale up of this tool [55], in addition to the fact that pit traps cannot be deployed in areas with a very low water table [32]. The comparison of pit traps to outdoor HLCs is indirect with regard mosquito behaviour, for instance, whilst HLCs target outdoor mosquito biting behaviour [13], pit traps target outdoor mosquito resting behaviour [20]. Pit traps are less likely to collect highly anthropophilic malaria vectors such as *An. funestus* that have been observed to bite outdoors in response to vector control [57–58]. This study shows that pit traps are a viable alternative to HLCs in sampling *Anopheles* vectors outdoors but did not provide accurate measures of transmission intensity. Pit traps, are relatively easy to set up, are very productive overall in terms of *Anopheles* vector density and assess a unique aspect of mosquito behaviour (outdoor resting) whose parameters are quite difficult to estimate [20, 31].

Whilst the choice for indoor/outdoor mosquito collection is most likely driven by entomologic measures of interest, HLCs provide measurements for both indoor and outdoor mosquito populations. Increased interest in mapping diurnal mosquito biting behaviour beyond night catches suggests that HLCs remain relevant [59]. Alternative indoor/outdoor collection methods including CDC light traps, prokopack aspirators and pit traps seem to be specialized mosquito collection methods targeting particular aspects of either indoor/outdoor HLCs. These aspects include, among others vector density, *Anopheles* species composition and sporozoite infection. As interest in alternative methods to HLCs gains momentum, some studies suggest using HLCs to calibrate mosquito collection measurements for alternative collection methods which can then be scaled up [9, 10, 60, 61]. This would in part address the challenges of overestimation of mosquito biting rates and EIR associated with CDC light traps [7]. As scalable tools, CDC light traps and prokopack aspirators present viable alternatives to HLCs indoors, however for outdoor sampling on a large scale, other alternatives such as the human baited double net method may need to be considered [62].

### Limitations

This study had several limitations. First, mosquito parameters such as parity, abdominal status and blood meal index, which may have provided additional granularity in the observed differences between trapping methods, were not measured. Second, not all indoor and outdoor alternatives were included. Alternative methods such as the human bait double net method and pyrethrum spray collections were not assessed due to resource limitations. Third, the study was limited to households located in 3 parishes within 2 districts in Eastern Uganda, and these findings may not be generalizable to other settings. Fourth, the houses used for CDC light trap collections were not the same as those used for other collection methods and variability in household characteristics was not accounted for. Finally, differences in the various methods, including the time period during which mosquitoes were collected and differences in targeting host-seeking vs resting mosquitoes, may have impacted on the results. Moreover, the data for this study were collected over only five months, not a complete calendar year, which may have affected aEIR estimates. Despite these limitations, the results of this study provide evidence on how alternative collection methods compare to HLCs to help guide future research studies and surveillance programmes.

## Conclusion

The method used to collect mosquitoes is important to consider when measuring entomologic outcomes and estimating transmission intensity. In this study, the density and species of mosquitoes collected with alternative methods varied, likely reflecting the feeding and resting characteristics of the common vectors and the different collection approaches. HLCs remain the gold standard for capturing host-seeking *Anopheles* mosquitoes indoors and outdoors during peak biting times, but the other methods evaluated have advantages. In this setting, CDC light traps provided a reasonable alternative to indoor HLCs, but prokopacks failed to collect a full representation of mosquitoes responsible for malaria transmission. Pit traps could be a useful alternative to HLCs for sampling outdoor resting mosquitoes, but mainly captured *An. arabiensis* and provided less accurate estimates of measures of transmission intensity. The potential impact of the method used to collect mosquitoes on the species composition of *Anopheles* collected and various entomologic endpoints should be carefully considered, particularly when assessing the effectiveness of vector control measures and estimating the impact on malaria transmission.

## Supplementary Information

**Additional file 1: Table S1. **Measures of association between method of collection and sporozoite infection, stratified by species.

## Data Availability

The data used are available from the corresponding author upon reasonable request.

## Abbreviations

LLIN: Long-lasting insecticidal nets
IRS: Indoor residual spraying
PCR: Polymerase chain reaction
EIR: Entomological inoculation rate
WHO: World Health Organization
HLC: Human landing catches
