# Supplementary material for: Impact of different mosquito collection methods on indicators of Anopheles malaria vectors in Uganda
Source: Malar J. 2022 Dec 19;21:388. doi: 10.1186/s12936-022-04413-1 (PMC9761930; doi:10.1186/s12936-022-04413-1)
Supplement: Supplementary file 1 — Additional file 1: Table S1. Measures of association between method of collection and sporozoite infection, stratified by species. [file 12936_2022_4413_MOESM1_ESM.docx]

**Additional Table 1: Measures of association between method of collection and sporozoite infection, stratified by species**

|  | ***Anopheles gambiae s.s.*** | | | | | ***Anopheles arabiensis*** | | | | | ***Anopheles funestus*** | | | | |
| --- | --- | --- | --- | --- | --- | --- | --- | --- | --- | --- | --- | --- | --- | --- | --- |
| **Indoor** | **N** | ***Pf***  **positive** | ***Pf* negative** | **SR (95% CI)** | **p-value** | **N** | ***Pf***  **positive** | ***Pf* negative** | **SR (95% CI)** | **p-value** | **N** | ***Pf***  **positive** | ***Pf* negative** | **SR (95% CI)** | **p-value** |
| HLC | 74 | 4 | 70 | 0.054 (0.0175-0.1399) | — | 203 | 1 | 202 | 0.005 (0.0003-0.0316) | **—** | 105 | 1 | 104 | 0.010 (0.0005-0.0595) | — |
| Prokopack | 59 | 0 | 59 | 0.000 (0.000-0.0762) | 0.15 | 26 | 1 | 25 | 0.038 (0.0020-0.2159) | 0.21 | 262 | 5 | 257 | 0.019 (0.0071-0.0465) | 0.68 |
| **Outdoor** |  |  |  |  |  |  |  |  |  |  |  |  |  |  |  |
| HLC | 62 | 0 | 62 | 0.000 (0.000-0.0727) | — | 125 | 1 | 124 | 0.008 (0.0004-0.0508) | **—** | 117 | 5 | 112 | 0.043 (0.0158-0.1018) | — |
| Pit trap | 49 | 1 | 48 | 0.020 (0.0011-0.1247) | 0.44 | 1036 | 4 | 1040 | 0.004 (0.0013-0.0106) | 0.43 | 123 | 0 | 123 | 0.000 (0.000-0.0377) | 0.03 |
